# Enterprise Privacy Resource Optimization and Big Data Intelligent Management Strategy Oriented to the Internet of Things

**DOI:** 10.1155/2022/7280695

**Published:** 2022-04-13

**Authors:** Bo Hou, Rong Huang

**Affiliations:** ^1^School of Economics and Management, Mianyang Teachers' College, Mianyang, Sichuan 621000, China; ^2^School of Accounting, Chongqing University of Technology, Chongqing 400052, China

## Abstract

In the context of the Internet of Things, user privacy leads to the lack of information security and the obscuration of traditional privacy concepts. Therefore, how to ensure information security and protect user privacy is a key issue that enterprises must solve in the process of using the Internet of Things technology. At present, the research on corporate employees' protection of user privacy in the context of the Internet of Things is mostly focused on the technical level, while the legal and management levels are relatively lacking. Based on the definition of the corporate concept in the context of the Internet of Things, this paper uses management and psychology as the research perspective, and based on theory of persuasion, adjustment orientation theory, and reinforcement theory, it discusses the attitudes of corporate employees to protect user privacy in the context of the Internet of Things and behaviour mechanism, constructing a new theoretical model. This experiment uses 0.001 as the step size to change the corresponding threshold size. The interval range is [0.001, 10], and there are a total of 10,000 points in the interval, which is equivalent to 100 million sensor attack tests. According to the above method, 10,000 points of the ROC curve can be obtained by using 10,000 thresholds, and the corresponding ROC curve can be drawn in the coordinate graph, which can intuitively reflect the performance of the VRADS vehicle anomaly real-time detection system. The challenge of data information protection is analyzed, trying to clarify the ideas for the protection of personal data and information in the Internet of Things environment and even lead to employees' rebellious psychology. This article proves that the pertinence and effectiveness of the persuasive content have a positive impact on employees' attitudes towards privacy protection, and it has been further deepened in the context of the Internet of Things. The balance point is to leave enough room for the long-term sustainable development of the Internet of Things industry on the basis of protecting the personal rights and interests of users.

## 1. Introduction 

The Internet of Things is the third wave of the world's information industry revolution after computers and the Internet [1]. It is gradually popularized in various fields such as military, industry, agriculture, power grid and water network, transportation, logistics, energy saving, environmental protection, medicine and health, and smart home, realizing the interconnection between any time, any place, and any object [2]. The Internet of Things is conducive to innovation management, identification and control, improving economic benefits, access and resource utilization, and realizing innovation in lifestyle and working methods. However, the information security of the Internet of Things is different from traditional network security. It is difficult for the existing technical solutions to effectively protect against security risks, and the user behaviour regulation and control is the key [3]. Therefore, exploring the attitudes and behaviours of enterprise employees to protect user privacy in the context of the Internet of Things from the perspectives of management and psychology is an innovation in the perspective and ways of protecting user privacy in the context of the Internet of Things. As an emerging network model, the Internet of Things is showing a trend of accelerated development worldwide. In recent years, different industries and different types of applications have gradually become popular and mature, and the world has entered a new era of Internet of Everything [4]. The “Internet of Things, Perceiving the World” feature of the Internet of Things is a “double-edged sword” which can not only enhance information sharing based on the smooth flow of information and realize the integration of human society and the physical environment, but also establish a closer logical connection of information [5].

The ubiquitous “smart objects” of the Internet of Things have comprehensive and accurate perception capabilities, the exposure of radio frequency identification attached to everyday objects, the highly transparent and information sharing of the Internet of Things, ubiquitous data perception, and wireless communication-based information [6]. The characteristics of transmission and intelligent information processing make it produce real-time and accurate location information. Therefore, the risk of privacy leakage increases with the tracking process of personal time and space, which is even higher than the information security in the traditional non-IoT context and privacy disclosure risks. Such risks will not only lead to the loss of information security, but also break through the boundaries of traditional privacy concepts [7]. Personal dignity will not be protected. In recent years, there has been a significant increase in the cases of companies disclosing user privacy. At present, with the advancement of Internet of Things technology in business operations, how to ensure information security and protect personal privacy are key issues that enterprises must solve in the context of Internet of Things. At present, most researches in this area concentrate on the technical level, and corporate employees' research on privacy disclosure and protection is blank, and it is difficult for existing technical solutions to effectively protect against security risks [8]. Therefore, from the perspectives of management and psychology, to strengthen corporate employees in the context of the Internet of Things, privacy protection research is very necessary and has important theoretical significance. This research introduces theory of persuasion, adjustment orientation theory, and reinforcement theory into this research, constructs a new theoretical model, and applies the above three theories to the attitudes and behavioural mechanisms of corporate employees towards user privacy protection in the context of the Internet of Things. The research has broadened the scope of application of the theory and realized the multiplication of theories [9]. At the same time, it has also expanded the research on privacy protection from the Internet field to the Internet of Things field. Through data analysis, the main factors that affect the privacy protection attitudes and behaviours of employees are found to provide scientific basis and suggestions for companies to formulate effective privacy protection policies in the context of the Internet of Things. It is helpful for companies to formulate effective privacy protection policies and contributes to the development of information security in our country. Even the construction of smart cities is of great significance [10].

This article aims to put forward the privacy protection attitude of employees in the context of the Internet of Things based on the particularity of the Internet of Things and the characteristics of the manager's persuasion process in the enterprise management process, combined with persuasion theory, adjustment orientation theory, and Skinner's reinforcement theory, and conduct empirical research on factors and models of behaviour and put forward relevant suggestions.

The first is the authority and credibility of the manager, the pertinence and effectiveness of the persuasive content, the fear of persuasive situation, and the positive strengthening of the relationship with the employee's privacy protection attitude; the second is the relationship between the employee's privacy protection attitude and the employee's privacy protection behaviour. The third is the moderating effect of the employee's prevention orientation on the relationship between the fear arousal of the persuasive situation and the privacy protection attitude, and the employee's promotion orientation has the moderating effect on the relationship between the positive reinforcement of the persuasion situation and the privacy protection attitude.

In the context of networking, the professionalism of privacy protection knowledge and the guidance of years of experience can make use of the influence of authoritative figures, give play to their knowledge, expertise, and leadership abilities, and have a strong influence on employee obedience.

In this research, objectives are as follows:

1. Discuss the privacy protection attitude and behaviour mechanism of employees in the context of the Internet of Things, construct a new theoretical model, and test the applicability of the mechanism model.
2. Test the relationship between all variables by using reliability analysis, validity analysis, correlation analysis, regression analysis, and other methods to verify the following relationships.

## 2. Related Work

Enterprises in the context of the Internet of Things are two different concepts from enterprises in the Internet of Things [11]. An Internet of Things enterprise refers to an enterprise that develops Internet of Things technology, and an enterprise in the Internet of Things situation refers to an enterprise that uses Internet of Things technology that may involve users' private information in their operations [12]. Enterprise privacy protection in the context of the Internet of Things is the act of protecting personal privacy in the context of the Internet of Things with technical, legal, and management means from leakage, tampering, and use by third parties in the context of the Internet of Things. This behaviour is beneficial to the protection of individuals or collectives, etc. Information that entities do not want to be known by outsiders ensures information security in the context of the Internet of Things. Enterprise privacy protection in the context of the Internet of Things has its unique properties: privacy in the Internet of Things includes identity privacy, data privacy, and location privacy, and this privacy information covers sensitive information about the environment, events, and objects that are closely related to daily life, with the reflection of the real needs of users; at the same time, personal information is constantly contacting and diverging due to needs and the expansion of user activities [13]. This is also the performance of the accuracy and reachability of privacy needs. This has led to companies satisfying people in the context of the Internet of Things. While providing convenient services, it runs counter to the goal of protecting personal privacy and brings new risks to information security [14].

Therefore, in the era of the Internet of Things technology, while strengthening technology and legislation, studying the protection of personal privacy from the perspective of sociology and psychology is a way that conforms to the nature of corporate privacy protection and has important innovative significance. The transparency of contextual information in the Internet of Things has led to an increase in the risk of leakage, and privacy in the Internet of Things is closely related to daily life [15]. This contradiction has led to an increase in the risk of privacy protection in the context of the Internet of Things. The characteristics of corporate privacy protection in the context of the Internet of Things are as follows: The difficulty of corporate privacy protection in the context of the Internet of Things [16]: The difficulty of protecting corporate privacy in the context of the Internet of Things is higher than that in the context of traditional Internet. In today's Internet of Things era, a high degree of transparency and sharing of information has become the biggest feature. However, the current widespread use of radio frequency technology and electronic tags has led to the universality of Internet of Things privacy leakage, which has broken through the traditional privacy rights. The concept has led to the difficulty of corporate privacy protection in the context of the Internet of Things compared to that in the traditional Internet context [17].

The vulnerability of corporate privacy protection in the context of the Internet of Things: In the context of the Internet of Things, corporate privacy leaks are concealed and difficult to detect. In the context of the Internet of Things, personal privacy information is universally sensitive information [18]. The sensitivity of information is combined with the inherent and common eavesdropping tag attacks for personal privacy, smart terminal loss or theft, eavesdropping attacks, and tampering attacks inherent in the Internet of Things. This situation increases the protection of corporate privacy in the context of the Internet of Things. It is impossible to estimate the degree of harm. In the context of the Internet of Things, corporate privacy protection lacks control [19]. In the application of the Internet of Things, the combination of identity information and geographic location has resulted in more user-sensitive information about the user's behaviour, habits, and consumption tendencies; at the same time, there is also privacy-inquired information in the query request [20]. In addition, with the development of information retrieval technology and search engine technology, many scattered information of users may also leak the privacy of users through information aggregation and association, and attackers can integrate various application systems through information retrieval technology [21]. In the end, users' sensitive privacy information is obtained, which results in the leakage of user privacy. However, a considerable part of the above privacy is unwilling to be disclosed by users.

## 3. A Theoretical Model of Corporate Privacy Protection and Behaviour Mechanism in the Context of the Internet of Things

### 3.1. Theoretical Model of Behaviour Mechanism

**Figure 1:**
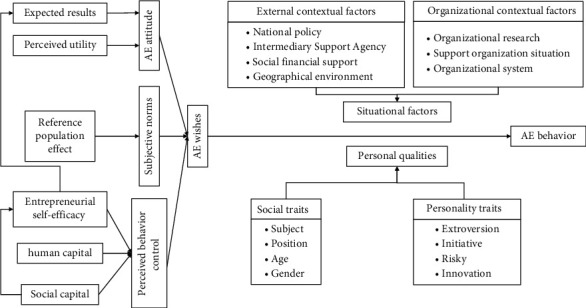
Theoretical model of behaviour mechanism.

On the basis of review and sorting, this paper applies theory of persuasion, adjustment orientation theory, and Skinner's reinforcement theory to construct a theoretical model of corporate employees' privacy protection attitudes and behaviour mechanisms in the context of the Internet of Things, as shown in Figure 1. Authority means that the identity, ability, and professional qualities of the persuader have the power and prestige to convince people, and they have high influence in the process of persuasion, enhancing the effectiveness of people's strong motivation and persuasive effect in pursuing the right choice. Credibility refers to the degree of trust and trust that the object of persuasion has on the persuader in terms of morality, motivation, purpose, or intention, and it has a huge impact on the persuasive effect. The essence of the manager's persuasion process is the process by which the manager conveys his ideas and values to his persuasion target—the employees. Only when the manager has the correct values and positive ideas can he increase reliability and honesty and correct the persuasive intentions. The source naturally states the correct views that conform to the mainstream and achieves the effect of “knowledge and action,” thereby strengthening the persuasive object's emotional tendency to trust, love, and identify with the manager and change the behaviour from the root. At the same time, it destroys the original state of information security and breaks through the traditional concept of personal privacy.

The combination of passiveness and powerlessness in information dissemination and control, as well as the undesirable consequences of privacy protection caused by the contradiction of the collection and use of information sent by individuals on these “human flesh terminals,” can make up for the existing Internet of Things as a new situation.

In empirical research, the development of the scale generally summarizes and sorts out the measurement items that have been widely used or repeatedly verified in relevant domestic and foreign literature and then makes corresponding modifications according to the research background. Based on the analysis and summarization of a large number of related documents, this article has formulated the measurement items for this study in combination with the characteristics of the enterprise in the context of the Internet of Things. The operation of each variable is shown in the following table, which also includes references for each variable corresponding to the item.

#### 3.1.1. Authority of Managers

Authority refers to the exemplary role and high influence of managers in actual work. The authority scale of managers is shown in Table 1.

#### 3.1.2. The Credibility of Managers

Credibility refers to the trust and trustworthiness of employees to managers, mainly by examining whether managers are credible in terms of morality, motivation, and intentions.

#### 3.1.3. Persuasion Content Pertinence

Pertinence refers to the fact that the content of privacy protection and persuasion can meet the needs of employees.

#### 3.1.4. Regulation Orientation of Employee Privacy Protection

Regulation orientation refers to the specific way or tendency of individuals in the process of self-regulation to achieve their goals. It is divided into promotion orientation and prevention orientation. Individuals with promotion orientation are proactive, and individuals with prevention orientation are relatively passive. The scale is shown in Table 2.

The analysis of this study used SPSS21.0 software to conduct descriptive statistics and inferential statistics. Details as follows:

1. Descriptive statistics: This study analyzes the demographic changes of the sample, sets the frequency distribution of the gender, age, working years, and industry of the employees of the Internet of Things technology that may involve user privacy in the business process, and calculates percentage, so as to understand the sample distribution.
2. Inferential statistics: using reliability analysis, validity analysis, correlation analysis, regression analysis, and other methods to analyze the reliability and validity of the scale, Spearman correlation analysis is used to test the relationship between all variables, and the regression analysis method verifies the following three groups of relationships. The first group of relationships: the authority and credibility of the manager, the pertinence and effectiveness of the persuasive content, the fear arousal and positive reinforcement of the persuasive situation, and the relationship between the employee's privacy protection attitudes; the second group of relationships: the employee's privacy protection attitude and the relationship between employees' privacy protection behaviours; the third group of relationships: the role of the employee's preventive orientation on the persuasive situation's fear arousal and the role of privacy protection attitudes.

### 3.2. Data Analysis

**Figure 2:**
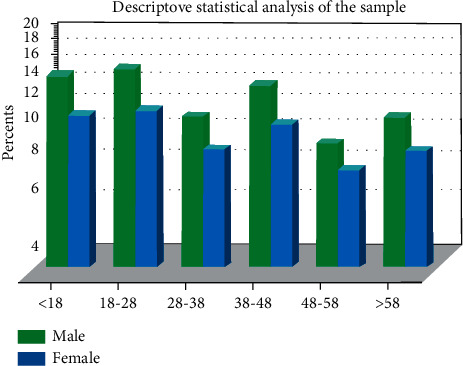
Descriptive statistical analysis of the sample.

Descriptive statistical analysis refers to descriptive statistics on the overall situation of the surveyed object to understand the overall situation of the surveyed person. 500 questionnaires were distributed in this survey, and 482 questionnaires were returned, with a recovery rate of 96.4%, of which there are 462 valid questionnaires, with an effective rate of 95.9%. SPSS 21.0 was used to perform descriptive statistical analysis on the recovered sample data, and descriptive statistics of the recovered sample. The analysis is shown in Figure 2.

The proportion of colleges and undergraduates is 84.9%, which is the absolute majority. The proportion of high school and below is only 6.5%, which shows that the employees who work in companies in the context of the Internet of Things generally have a higher level of education.

Reliability analysis refers to the consistency or stability analysis of the measurement method, mainly to check whether the compiled scale has consistency and stability when measuring various variables. Only when a measurement model has a high degree of reliability can its results have reference value. The most commonly used index for evaluating internal consistency reliability is Cronbach's *α*. The larger the value of Cronbach's *α*, the stronger the correlation between the measurement items. Cronbach's *α* value is generally required to reach at least 0.7; item—the overall correlation coefficient (CITC) is greater than 0.5; in addition, if deleting an item will significantly increase the Cronbach' *α* of the variable, the item should also be deleted item. We used SPSS21.0 statistical software to test Cronbach's *α* value of each dimension of self-service technical quality and the item of each measurement item—the overall correlation coefficient (CITC).

### 3.3. Big Data Intelligent Management Strategy

**Figure 3:**
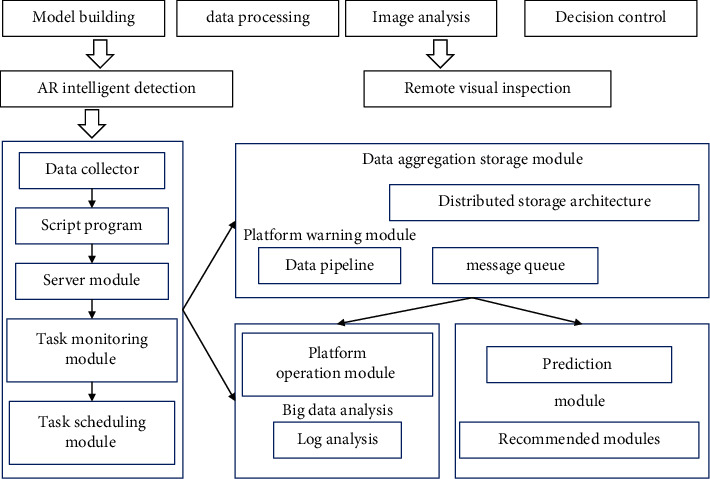
Big data intelligent management strategy.

The authority of managers has a negative impact on employees' privacy protection attitudes (H1a fails the hypothesis test), and the trustworthiness of managers has no significant positive impact on employees' privacy protection attitudes (H1b fails the hypothesis test). These sensors are likely to become merchants “monitoring” and “location” tool, without people's knowledge, constantly digging up users' personal information, which greatly threatens users' personal privacy rights; in addition, advanced technology makes privacy violations intelligent, leading to the definition of privacy protection issues. The complexity of personal privacy, the relevance of personal privacy content and daily life, the nature of humans to snoop on privacy, the relatively weak awareness of privacy protection of Chinese citizens, and the backwardness of privacy protection in our country have led to the popularization of the Internet of Things technology that has broken the boundaries of traditional privacy. People's privacy poses a great challenge, as shown in Figure 3. In particular the combination of the Internet of Things, the characteristics of the world's perception, the ubiquity of devices, and the characteristics of human prying privacy have enhanced the complexity of privacy protection management. It will hinder the influence of the authority and credibility of the manager on the user's privacy protection attitude to a certain extent and affect the significance of the original assumption.

In this research, the pertinence of persuasive content has a positive impact on employees' attitudes towards privacy protection (H2a passes hypothesis testing). To make the content of persuasion pertinent, it is necessary to make the content meet the reality of the persuasion situation and the needs of the persuader and make the manager's persuasion information and concepts practical and precise and meet the needs of the persuaded object.

Kalman filter uses linear minimum variance estimation, the first and second moments of the known estimator *X* and the observation *Z*, namely, the mean *E*[*X*], *E*[*Z*] and variance VAR[*X*], VAR[*Z*] and in the case of covariance COV[*Z*, *X*], it is assumed that the estimator sought is a linear function of the observables, and the estimation method of the performance index (loss function) of the optimal estimation is the minimum of the estimated error variance matrix. Assuming that the linear function of the observation *Z* is estimated to be *X*, then set

(1) $$\begin{array}{c} E\left[X\right]=a+bE\left[Z\right]. \end{array}$$

In the formula, *B* is a nonrandom matrix; the number of rows and columns is consistent with the dimensions of *X* and *Z*, respectively; *A* and *X* have the same dimension. By definition, the minimum variance estimate *X*(*Z*) is

(2) $$\begin{array}{c} X\left(Z\right)=E\left[X\right]+COV^{-1}\left[Z,X\right]\left(Z-E\left(x\right)\right). \end{array}$$

Assuming that *X*(*k*) is the n-dimensional vector of the target state at time *n*, *h*(*k*) is the *h* state transition matrix, *G*(*k*) is the input matrix, *u*(*k*) is the input vector, *w*(*k*) is the process Gaussian white noise, then the state prediction equation is

(3) $$\begin{array}{c} X\left(k+1\right)=a\varphi \left(k\right)+b\varphi^{-1}\left(k\right). \end{array}$$

Satisfaction is in the formula

(4) $$\begin{array}{c} X\left(k+1\right)=0;\varphi \left(k\right)=c. \end{array}$$

Suppose the signal measurement equation is

(5) $$\begin{array}{c} \varphi \left(k\right)=c=a-bk, \end{array}$$

where *h*(*K*) is the measurement matrix and *V*(*K*) is the measurement noise, which satisfies

(6) $$\begin{array}{c} E\left(V\left(K\right)\right)=V_{{}^{T}}\left(j\right)V\left(j\right). \end{array}$$

Assuming that the filter value at time *k* is *X*(*k*), and the covariance matrix is *p*(*k*), the state prediction value can be obtained through the Kalman algorithm, namely:

(7) $$\begin{array}{c} X\left(\frac{k+1}{k}\right)=\varphi \left(k\right)+\frac{G\left(k\right)}{p\left(k\right)}. \end{array}$$

The above is a Kalman filter model based on a linear mathematical model. The state equations and measurement equations of the standard Kalman filter are linear models. It is not theoretically possible to find a strict filter recursive equation in a nonlinear system, and most of its solution ideas are described by an approximate method; that is, the linearization of the nonlinear system is used for further research.

In the research of this article, the persuasive content of privacy protection in the context of the Internet of Things should be more in line with the high risk of information disclosure caused by the inherent characteristics of the Internet context, the severity of the consequences of losses, and the nature of people to spy on the privacy of others, and individual control capabilities. The shortcomings of the content of persuasion can make the content of persuasion achieve good results. In this study, the effectiveness of persuasive content has a positive impact on employees' attitudes towards privacy protection (H2b passes hypothesis testing). Persuasion content and the effectiveness of persuasion means are the essential components of the persuasive effect, and it is also the breakthrough point for the persuasion content to realize the change in the attitude of persuasion. The change of the persuader's attitude depends on the effective content of persuasion to penetrate the persuader's active and passive defenses. Therefore, the null hypothesis has been verified, and the significance level is high.

## 4. Resource Optimization Adjustment

Among the population participating in the questionnaire survey, men accounted for 55% and women accounted for 45%, which is basically close to a 1 : 1 ratio. At the academic level, it is dominated by people with higher education. Among them, the proportion of undergraduates and masters and doctors is as high as 93.5%.

**Figure 4:**
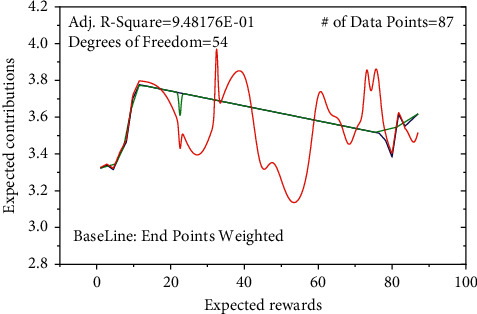
Changes in repetitive enhancement mechanism.

The fear of persuasive situation has a positive effect on employees' privacy protection attitude (H3a passes hypothesis test). The fear arousal of persuasive situation is a concrete manifestation of the application of deterrence theory in the field of information security and communication. Deterrence, as a means of formal control by an organization, is embodied in the certainty and severity of punishment. The main point of this theory is as people perceive the certainty and severity of punishment to increase, illegal behaviours will decrease, namely, unwanted behaviour can be deterred by the threat of punishment, and fear of punishment can prevent violations. Perceived sanctions and threat assessment have a positive impact on employees' intention to comply with Internet use strategies. Therefore, the null hypothesis passes the test and has good significance. In this study, the positive reinforcement of the persuasive situation has a positive impact on employees' attitudes towards privacy protection (H3b passes hypothesis testing). From the perspective of reinforcement theory, reinforced behaviour tends to recur after reinforcing the “consequence” of the possibility of recurring in the future, as shown in Figure 4. From the perspective of positive reinforcement, rewards, as a mechanism for the organization to motivate employees to follow the Internet use strategy, are also a recognition of the employees' compliance behaviour and organizational goals. It also tells employees what behaviour is advocated by the organization in another way and what behaviour is not allowed by the organization; therefore, rewards have a positive impact on employees' intention to comply with the Internet usage strategy. Expected rewards, expected relationships, and expected contributions have a positive and significant impact on behaviour and attitudes.

**Figure 5:**
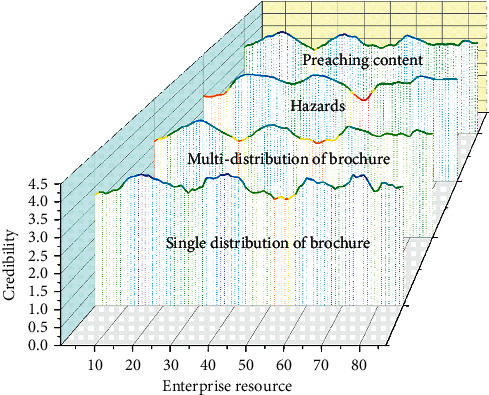
Changes in credibility of enterprise resource optimization.

From the perspective of managers, they should further strengthen their authority and credibility and establish prestige, that is, correct the leader's majestic image, enrich the professional knowledge of privacy protection in the context of the Internet of Things, accumulate management experience, and give play to their knowledge, expertise, and guidance ability to enhance radiation and scientific management, establish a bottom line for employees' behaviour to protect user privacy, and strengthen employees' motivation to protect user privacy. In enterprise management, the privacy protection of users in the context of the Internet of Things is not only a technical issue, but also a social issue. Especially for the context of the Internet of Things in this article, if you want to successfully realize the privacy protection of the Internet of Things, you must consider the Internet of Things. With the characteristics of high degree of information transparency and information sharing, ubiquitous data perception, wireless communication-based information transmission, and intelligent information processing, coupled with the nature of people to snoop on privacy and mobile applications based on smart phones, there is a risk of leaking user privacy. Managers should further strengthen the correct information security values, led by example, accumulate professional knowledge and management experience, improve management capabilities, and establish authority and reliability. Credibility: Enterprises with conditions can hire relevant experts to guide users' privacy protection in the context of the Internet of Things of their enterprises, as shown in Figure 5.

**Figure 6:**
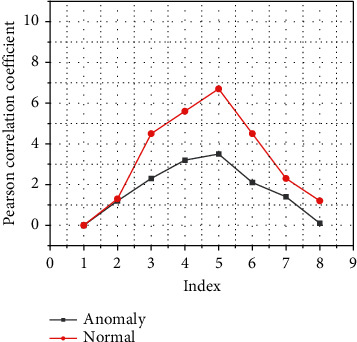
Pearson correlation coefficient corresponding to Corr7 in the correlation circle.

In this experiment, according to the detection process of the VRADS automobile anomaly real-time detection system, first calculate the corresponding nodes (the automobile inertial measurement unit is in the Pearson correlation coefficient between automobile directional acceleration and wheel torque, that is, Corr7 in the correlation ring, the real-time curve of its change is shown in Figure 6, and the corresponding Pearson is calculated in units of 1 sliding window. Correlation coefficient: Among them, the change of Corr7 in the correlation loop under normal conditions is shown in Figure 6; under normal conditions, the Pearson correlation coefficients between the corresponding sensor variables are all higher than 0.9. However, from the changes in the Pearson correlation coefficient of the abnormal data indicated by the red line in the figure, the following conclusions can be drawn. When the car has an abnormal situation near the 13400th sample point, the Pearson correlation coefficient also has a sudden change, and it is abrupt. If it falls below 0.5, it means that the sharp shape formed by this abrupt falling curve can represent an abnormality in the car. This fully shows that, in the sensor hijacking attack scenario represented by Scenario A, the VRADS car anomaly real-time detection system uses the characteristics of rapid decline in the correlation in the car's multisource sensor data and can effectively detect the car's anomaly in time.

**Figure 7:**
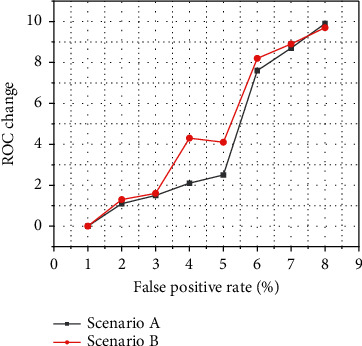
ROC change curve of abnormal real-time detection system performance.

Similar experimental simulation verification can also be completed in the abnormal attack scenarios represented by Scenario B and Scenario C, so we will not repeat them here. Based on the above analysis, it can be concluded that the VRADS vehicle anomaly real-time detection system can effectively distinguish the abnormal and normal state of the vehicle through the correlation change between the vehicle's multisource sensor variables, so the correlation coefficient between the multisource sensor variables is used. It is completely effective and feasible to perform real-time detection of car abnormalities based on changing conditions and as a criterion for judging whether the car is in an abnormal state. This experiment uses 0.001 as the step size to change the corresponding threshold size. The interval range is [0.001, 10], and there are a total of 10,000 points in the interval, which is equivalent to 100 million sensor attack tests. In each attack test, this experiment randomly selects the attack node from the correlation ring and uses the aforementioned strategy to randomly tamper with the 300 sample data in the corresponding node and then further according to the corresponding threshold determine whether the car is abnormal, and finally count at the threshold. The VRADS vehicle anomaly real-time detection system under VRADS detects the true positive rate and false positive rate of vehicle anomalies, thereby obtaining the position of one of the coordinate points of the ROC curve, as shown in Figure 7. According to the above method, 10,000 points of the ROC curve can be obtained by using 10,000 thresholds, and the corresponding ROC curve can be drawn in the coordinate graph, which can intuitively reflect the performance of the VRADS vehicle anomaly real-time detection system.

From the perspective of the content of persuasion, in the daily management content of the enterprise, more attention should be paid to the effectiveness of the content of persuasion, and more attention should be paid to the new characteristics of the Internet of Things situation to formulate corporate privacy protection policies. In the context of this article, the characteristics of the Internet of Things increase the risk of privacy leakage. Therefore, in daily management, the privacy protection content should be instilled in the context of the Internet of Things, and the persuasion should be strengthened; in addition, the needs of different types of employees should be taken into consideration. Take employees as the carrier, understand employee psychology and specific management environment, and find effective and targeted persuasion methods and strategies based on employee characteristics; at the same time, consider employees' first contact with the Internet of Things and lack of experience, using easy-to-understand terms. Specifically, the further application of the theory of deterrence widely used in the field of information security is crucial for the universality and pervasiveness of privacy leaks and the passivity of individual control capabilities in the context of the Internet of Things. Therefore, when managers set up persuasive situations, they should increase penalties and use statistical data, pictures, videos, and other methods to show employees the hazards of privacy protection in the context of the Internet of Things and should implement punitive measures. In terms of positive reinforcement, the good effect of employees in protecting user privacy is linked to rewards, wages and benefits, and vacation benefits, which has a good incentive effect.

## 5. Conclusion

This thesis studies the influencing factors of corporate employee privacy protection in the context of the Internet of Things from the perspectives of management and psychology. Based on literature review and related theories, through the analysis of the manager's persuasion process to employees, it summarizes the impact on the enterprise in the context of the Internet of Things. The direct factors of employee privacy protection, through empirical analysis, verifying theoretical models, drawing conclusions, and explaining, provide a new perspective for companies to strengthen user privacy protection research in the context of the Internet of Things and have a certain innovative value. This article analyzes the influence of managers' authority and credibility factors on employees' attitudes towards privacy protection, and the results are as follows: In the business process involving IoT applications, people's privacy and preferences, as well as the exposure of information, will be affected. The influence of the authority and credibility of the manager has begun to weaken; in terms of the authority of the manager, although the authority of the manager means an exemplary role and high influence, it sometimes becomes a kind of pressure on the employees and even leads to employees' rebellious psychology. This article proves that the pertinence and effectiveness of the persuasive content have a positive impact on employees' attitudes towards privacy protection, and it has been further deepened in the context of the Internet of Things. Persuading content that is relevant to the Internet context is actually the most effective means to enhance the persuasive effect. In particular, it should strengthen the persuasive content of the universality of corporate user privacy disclosure and the vague concept of privacy in the context of the Internet of Things and enhance its effectiveness. These all reflect that the positive reinforcement of the persuasive situation is positively related to the employee's privacy protection attitude, and the original assumptions have been verified.

## Figures and Tables

**Table 1:** Authority scale for managers.

Manager	Item	Source
AU1	Protect user privacy	Credibility
AU2	Demonstrate the correct way to protect user privacy	Feasibility
AU3	Protect user privacy expertise	Credibility
AU4	Protect user privacy and professional quality	Feasibility
AU5	Give priority to high-impact pictures	Feasibility

**Table 2:** Precautionary vector table for privacy protection.

Manager	Item	Classification
AU1	Avoid leakage of user privacy	Privacy protection attitude
AU2	Responsibilities and obligations	Preventive orientation
AU3	Maintain information security	Promote orientation
AU4	Protect user privacy and professional quality	Positive reinforcement
AU5	The goal of protecting user privacy	Fear arouses

## Data Availability

The data used to support the findings of this study are available from the corresponding author upon request.

## References

[1] Agyabeng-Mensah Y., Ahenkorah E., Afum E., Nana Agyemang A., Agnikpe C., Rogers F. (2020). Examining the influence of internal green supply chain practices, green human resource management and supply chain environmental cooperation on firm performance. *Supply Chain Management: International Journal*.

[2] DeGregorio G. Technology Management via a Set of Dynamically Linked Roadmaps.

[3] Dhamija P., Bag S. (2020). Role of artificial intelligence in operations environment: a review and bibliometric analysis. *The TQM Journal*.

[4] Ghobakhloo M., Fathi M. (2019). Corporate survival in Industry 4.0 era: the enabling role of lean-digitized manufacturing. *Journal of Manufacturing Technology Management*.

[5] Grant E. (2021). Big data-driven innovation, deep learning-assisted smart process planning, and product decision-making information systems in sustainable industry 4.0[J]. *Economics Management and Financial Markets*.

[6] Humayun M., Jhanjhi N., Alruwaili M., Amalathas S. S., Balasubramanian V., Selvaraj B. (2020). Privacy protection and energy optimization for 5G-aided industrial Internet of Things. *IEEE Access*.

[7] Kumar N., Kumar G., Singh R. K (2021). Big data analytics application for sustainable manufacturing operations: analysis of strategic factors. *Clean Technologies and Environmental Policy*.

[8] Kumar M. S., Raut D. R. D., Narwane D. V. S., Narkhede D. B. E. (2020). Applications of industry 4.0 to overcome the COVID-19 operational challenges. *Diabetes & Metabolic Syndrome: Clinical Research Reviews*.

[9] Li C., Chen Y., Shang Y. (2021). A review of industrial big data for decision making in intelligent manufacturing. *Engineering Science and Technology, An International Journal*.

[10] Patwa N., Sivarajah U., Seetharaman A., Sarkar S., Maiti K., Hingorani K. (2021). Towards a circular economy: an emerging economies context. *Journal of Business Research*.

[11] Phaal R., Muller G. Towards visual strategy: an architectural framework for roadmapping.

[12] Raut R. D., Gotmare A., Narkhede B. E., Govindarajan U. H., Bokade S. U. (2020). Enabling technologies for industry 4.0 manufacturing and supply chain: concepts, current status, and adoption challenges. *Ieee Engineering Management Review*.

[13] Raut R. D., Mangla S. K., Narwane V. S., Gardas B. B., Priyadarshinee P., Narkhede B. E. (2019). Linking big data analytics and operational sustainability practices for sustainable business management. *Journal of Cleaner Production*.

[14] Rojko A. (2017). Industry 4.0 concept: background and overview. *International Journal of Interactive Mobile Technologies (iJIM)*.

[15] Seuring S., Müller M. (2008). From a literature review to a conceptual framework for sustainable supply chain management. *Journal of Cleaner Production*.

[16] Shamim S., Cang S., Yu H., Li Y. Management approaches for Industry 4.0: a human resource management perspective.

[17] Sivarajah U., Kamal M. M., Irani Z., Weerakkody V. (2017). Critical analysis of Big Data challenges and analytical methods. *Journal of Business Research*.

[18] Tsolakis N., Niedenzu D., Simonetto M., Dora M., Kumar M. (2021). Supply network design to address United Nations Sustainable Development Goals: a case study of blockchain implementation in Thai fish industry. *Journal of Business Research*.

[19] ur Rehman M. H., Yaqoob I., Salah K., Imran M., Jayaraman P. P., Perera C. (2019). The role of big data analytics in industrial Internet of Things. *Future Generation Computer Systems*.

[20] Wang G., Gunasekaran A., Ngai E. W. T., Papadopoulos T. (2016). Big data analytics in logistics and supply chain management: certain investigations for research and applications. *International Journal of Production Economics*.

[21] Waris M. M., Sanin C., Szczerbicki E. (2016). Smart innovation management in product life cycle. *Advances in Intelligent Systems and Computing*.

